# Supplementary material for: Postnatal cytomegalovirus infection and bronchopulmonary dysplasia in very low birth weight infants: Influence of diagnostic criteria and viral load in a propensity score–matched cohort study
Source: PLoS One. 2026 Apr 15;21(4):e0346905. doi: 10.1371/journal.pone.0346905 (PMC13082651; doi:10.1371/journal.pone.0346905)
Supplement: Attachment — Submitted filename: Response to Reviewers.docx [file pone.0346905.s003.docx]

**Response Letter**

**Manuscripts number:** PONE-D-25-54667

**Title:** Postnatal cytomegalovirus infection and bronchopulmonary dysplasia in very low birth weight infants: Influence of diagnostic criteria and viral load in a propensity score-matched cohort study

Dear Editors and Reviewers,

We appreciate the opportunity to revise our manuscript titled "Postnatal cytomegalovirus infection and bronchopulmonary dysplasia in very low birth weight infants: Influence of diagnostic criteria and viral load in a propensity score-matched cohort study" and are grateful for the insightful comments provided by the reviewers. Those comments are all valuable and very helpful for revising and improving our paper, as well as the important guiding significance to our researches. In the following section, we provid detailed responses to each comments. Specifically, the comments are presented in *italics*, our responses are shown in red, and changes to the manuscript are marked in yellow.

*Journal Requirements:*

*When submitting your revision, we need you to address these additional requirements.*

*1. Please ensure that your manuscript meets PLOS ONE's style requirements, including those for file naming. The PLOS ONE style templates can be found at*

*https://journals.plos.org/plosone/s/file?id=wjVg/PLOSOne_formatting_sample_main_body.pdf and*

*<https://journals.plos.org/plosone/s/file?id=ba62/PLOSOne_formatting_sample_title_authors_affiliations.pdf>.*

Response：We appreciate the reminder. We have meticulously examined the PLOS ONE style templates and have revised the manuscript to ensure complete adherence to all formatting and style requirements. Additionally, we have adhered to the specified file naming conventions.

*2. Thank you for stating the following financial disclosure:*

*“National Key Research and Development
Program of China, Grant/Award Number:
2022YFC2703500; 4+X Clinical Research
Project of Women's Hospital, School of
Medicine, Zhejiang University,
Grant/Award Number: ZDFY2021‐4X205”*

*Please state what role the funders took in the study.  If the funders had no role, please state: "The funders had no role in study design, data collection and analysis, decision to publish, or preparation of the manuscript."*

*If this statement is not correct you must amend it as needed.*

*Please include this amended Role of Funder statement in your cover letter; we will change the online submission form on your behalf.*

Response：Thank you for the reminder. It is important to note that the funding entities did not influence the study's design, data collection and analysis, decision to publish, or the preparation of the manuscript. As per your request, this statement has been incorporated into the cover letter.

*3. We note that the grant information you provided in the ‘Funding Information’ and ‘Financial Disclosure’ sections do not match.*

*When you resubmit, please ensure that you provide the correct grant numbers for the awards you received for your study in the ‘Funding Information’ section.*

Response：We appreciate your identification of the inconsistency in the grant information. We have conducted a thorough review and made the necessary corrections to ensure alignment between the 'Funding Information' and 'Financial Disclosure' sections.

4. *Please ensure that you include a title page within your main document. You should list all authors and all affiliations as per our author instructions and clearly indicate the corresponding author.*

Response：We appreciate your reminder. In accordance with the journal's author guidelines, we have included a dedicated title page in the revised manuscript. This title page enumerates all authors and their full affiliations. The corresponding author is distinctly identified, with their contact information, including email and postal address, provided.

5. *Please amend your manuscript to include your abstract after the title page.*

Response：We appreciate your bringing this matter to our attention. In response, we have revised the manuscript to position the abstract following the title page, in accordance with the specified requirements.

6. *Please include a separate caption for each figure in your manuscript.*

Response：We appreciate your bringing this matter to our attention. We have revised the manuscript to include distinct descriptive captions for each figure, ensuring that each caption clearly articulates the content and significance of the corresponding figure.

7. *Please include your tables as part of your main manuscript and remove the individual files. Please note that supplementary tables (should remain/ be uploaded) as separate "supporting information" files*

Response：Thank you for your reminder. We've revised the manuscript to incorporate all essential tables directly into it, removing their separate files.

8. *If the reviewer comments include a recommendation to cite specific previously published works, please review and evaluate these publications to determine whether they are relevant and should be cited. There is no requirement to cite these works unless the editor has indicated otherwise.*

Response：We appreciate your notification regarding the editorial policy. We acknowledge receipt of this policy and affirm our commitment to complying with its stipulations. Furthermore, we confirm that, in the current review round, none of the reviewers suggested the citation of specific publications. Consequently, this policy does not pertain to the present revisions.

*Reviewer #1: Thank you for the opportunity to review the manuscript entitled “Postnatal cytomegalovirus infection and bronchopulmonary dysplasia in very low birth weight infants: influence of diagnostic criteria and viral load in a propensity score-matched cohort study”. This study addresses an important and clinically relevant issue in neonatal intensive care-the association between pCMV infection and BPD in VLBWIs. However, several major methogological and interpretive concerns should be addressed before its being considered for publication.
1.The manuscript needs careful writing editing. Many sentences contain grammatical mistakes and wrong sentence structure, which make the article difficult to understand.*

Response：We sincerely appreciate the reviewer's valuable feedback. We polished the language by the editing service to improve readability of the manuscript. The text has been carefully edited by a professional English language editor to ensure clarity and readability. We have also double-checked the entire manuscript to eliminate any remaining language issues.

*2.Introduction: Necessary references need to be added to the concept of cCMV and pCMV.*

Response：We have added concept and reference to definite congenital CMV (cCMV) and postnatal CMV

(pCMV) in the introduction section：Congenital CMV (cCMV) infection is diagnosed via polymerase chain reaction (PCR) or viral culture of urine samples, respiratory tract secretions, blood, or cerebrospinal fluid obtained within the first 3 weeks of life. Postnatal cytomegalovirus (pCMV) infection is defined as a cytomegalovirus infection after 3 weeks of age upon the exclusion of cCMV infection (P4 L65-69)

The added references :

[8] Leruez-Ville M, Chatzakis C, Lilleri D, Blazquez-Gamero D, Alarcon A, Bourgon N, et al. Consensus recommendation for prenatal, neonatal and postnatal management of congenital cytomegalovirus infection from the European congenital infection initiative (ECCI). The Lancet Regional Health – Europe. 2024;40. doi:10.1016/j.lanepe.2024.100892

*3.Study population: I’m confused with the inclusion and exclusion criteria. What’s the protocol for CMV screening in the unit? Are there any changes in CMV screening during the 11-year period? When was CMV testing done for these pCMV infants?*

Response : We appreciate the reviewer's concern regarding the CMV screening protocol. In our study, the CMV screening protocol remained consistent throughout the 11-year period and added in the manuscript：

To rule out cCMV, all preterm infants were routinely screened via urine and respiratory secretion samples within the first 48–72 h and repeated 2–3 weeks after birth. A pCMV infection was diagnosed when testing was prompted by clinical suspicion beyond 3 weeks of age based on relevant symptoms (e.g., sepsis-like syndrome, gastrointestinal manifestations) or laboratory abnormalities (e.g., thrombocytopenia, transaminitis).(P6 L94-98)

*4.Results: There is no need to repeat the data in the results in text, which have been shown in the tables very clearly. Are there any infants received Ganciclovir therapy?*

Response : Thank you for the suggestion. We've streamlined the Results section by removing repetitive descriptions of numerical data already shown in the tables. The text now emphasizes key trends, highlights significant findings, and offers interpretation, directing readers to the tables for specific values.

In our cohort, 28/74 (37.8%) infants with postnatal cytomegalovirus (pCMV) infection were administered ganciclovir therapy. To account for this variable, we incorporated "ganciclovir treatment (yes/no)" as a covariate in the fully adjusted Model 5 of our regression analysis. This adjustment has been explicitly described in the Statistical Analysis (P7 L129 )and Results sections(P14 L171) of the revised manuscript.

*5. Discussion: There is logistic problem in the first sentence of the last paragraph on Page 7. “but the 2001 NICHD standard shows significant differences in stratified diagnosis”, why “but” here?*

Response : Thank you for pointing out the logical inconsistency. We have revised the sentence to clarify the intended contrast. The revised sentence now reads: Our study found significant differences in the 2018 NICHD and 2019 NRN criteria between pCMV-infected and non-pCMV-infected groups in the overall incidence and Grades II–III/2–3. In contrast, the 2001 NICHD criteria revealed significant differences between the moderate and severe groups only. However, the overall diagnostic rate did not differ significantly between groups.(P21 L225-228)

*6.The discussion on the high viral load and BPD is rather general and far fetched. More evidence should be provided to demonstrate the relationship between high CMV load and severity of BPD.*

Response :We appreciate the reviewer's constructive feedback. In the revised manuscript, we have strengthened the discussion by including additional references and data，these additions can be found in the discussion sections.

Our findings indicate that an elevated CMV viral load (≥1.34 × 10⁵ copies/mL) is associated with an increased incidence of BPD as well as worsened respiratory outcomes, prolonged IMV duration, and extended respiratory support in VLBW infants (Table 5). This aligns with prior reported that a high CMV DNA load (>50,000 copies/mL) was independently associated with organ hemorrhage (OR, 3.61; 95% CI, 1.03–12.65) [37]. Additionally, a higher viral load has been correlated with more severe manifestations and poorer outcomes in cases of CMV pneumonia among immunocompetent hosts and CMV disease in transplant recipients [38, 39]. Although specific thresholds have not been established, a higher viral load is associated with symptomatic infection and poor prognosis [40–42]. The potential mechanisms by which a high CMV load exacerbates BPD are multifaceted. First, elevated viremia can trigger a systemic inflammatory response or viral sepsis characterized by the release of proinflammatory cytokines [43]. This systemic inflammation may exacerbate the inflammatory environment in the developing preterm lung, thereby disrupting alveolarization and promoting fibrosis, the key features of BPD pathogenesis. Second, the CMV induces endothelial injury and dysfunction [44]. Within the pulmonary vasculature, this can impair angiogenesis and vascular remodeling, further worsening BPD severity. Finally, human CMV (HCMV) infection is typically managed by a robust immune response; however, in individuals with compromised immune systems, HCMV can replicate at high levels, leading to end-organ disease (EOD) [45]. In compromised hosts, particularly neonates who are preterm or have a VLBW, the absence of T cell and natural killer (NK) cell function facilitates unrestricted viral replication. This results in high viral loads and ultimately culminates in EOD, which is characterized by direct viral damage, such as retinitis and pneumonia, and indirect effects, including endothelial injury [46,47]. However, prior research predominantly concentrated on cCMV infections; thus, the relationship between viral load and disease severity in pCMV infections requires further exploration. (P21-22 L244-265)

*7.What are the possible infection routes for these pCMV infants?*

Response : Thank you for your question, it is posited that the primary mode of transmission is through exposure to breast milk. Due to the long time span, much data of breast milk was missing and not included in the discussion. We addde the possible infection routes in the passage: Possible routes of pCMV infection in infants include breast milk, blood products, and horizontal transmission. In the current era, with the reduction in CMV transmission via blood products achieved through seronegative donor selection and leukoreduction, exposure to breast milk from CMV-seropositive mothers has emerged as the most common transmission route.(P4-5 L69-72)

*8.In the limitations the author mentioned “the control group comprised both untested infants and those with negative test results”. I think there is a high probability of bias here, affecting the credibility of the results.*

Response :

We appreciate the reviewer's concern regarding potential bias in the control group composition. We acknowledge this as a limitation in the study design. The absence of universal CMV screening could lead to misclassification bias, potentially resulting in the omission of asymptomatic pCMV infections within the control group. Importantly, such non-differential misclassification is expected to bias the findings towards the null hypothesis, thereby underestimating the true effect size. Consequently, the significant associations we have reported likely represent conservative estimates. We will address and clarify this point in the revised Limitations section of the manuscript: Although this method aligns with real-world clinical practice and is prevalent in the literature [23,51], it introduces a potential misclassification bias. Therefore, some asymptomatic pCMV infections may have been overlooked in the control group. This non-differential misclassification is likely to bias our findings toward the null hypothesis by attenuating the intergroup differences. Consequently, the observed associations between pCMV infection and BPD, particularly when newer diagnostic criteria were applied, are likely conservative estimates of the true effect. (P23 L274-279)

*9.There are also a lot of formatting issue in the tables.*

Response :We have carefully reviewed all tables in the manuscript and corrected the formatting issues. The tables have been standardized to ensure consistent font size, alignment, and spacing throughout the document. Additionally, we have ensured that all table headers are clearly labeled and that the data is presented in a clear and organized manner.

*Reviewer #2: Thank you for the opportunity to review "postnatal cytomegalovirus infection and bronchopulmonary dysplasia in very low birth weight infants: influence of diagnostic criteria and viral load in a propensity score matched cohort study." The authors ask a novel study question of if postnatal CMV infection is associated with 3 clinically distinct definitions of BPD using propensity score matching and hypothesize that an association will be identified. The authors found that the association of pCMV infection with BPD was influenced by diagnostic criteria of BPD and that higher viral load was associated with more severe respiratory morbidity. These findings have biological plausibility and represent a potentially modifiable risk factor for BPD. Thank you for addressing my concerns.

Major concerns:
1) Matching was performed using GA, BW, SGA, sex, and birth year; however, even after matching, infants with pCMV had higher rates of late-onset sepsis, transfusion, steroid use, diuretic exposure, and longer respiratory support. These differences likely reflect underlying illness severity, which may simultaneously increase the probability of CMV testing and the risk of BPD. This limitation should be acknowledged more explicitly, and conclusions should avoid overstating causality. These imbalances also suggest notable residual confounding and potentially incomplete control of early illness severity. Did the authors consider using a more robust 1:2, 1:3, or 1:4 match? This more robust approach may improve the match and would strengthen the results. Alternatively, the authors could exact match on GA week, BW category/SGA status, sex, birth year, and use propensity score matching on a number of other variables to improve the causal inference of the study. In any case, the authors should provide balance diagnostics (i.e., standardized mean differences before and after matching) for the cohort to help the orient the reader to the strength of the results.*

**Response:**

We are grateful to the reviewer for this crucial methodological point. We concur on the significance of controlling for confounding variables and evaluating the quality of matching; accordingly, we have endeavored to address these issues within the limitations imposed by the available data.

We carefully consider the reviewer's suggestion for 1:2, 1:3, or 1:4 match or exact match on other variables. However, expanding the ratio would have potentially introduced greater heterogeneity without a guaranteed improvement in balancing unmeasured factors. Given the strict matching criteria, the pool of highly comparable uninfected controls was limited. We agree that providing the standardized mean difference (SMD) before and after matching is an ideal way to assess the matching effect. As suggested, we now provide the Standardized Mean Differences (SMD) in the revised Table 2, which show balance (SMD <0.2) for most matching variables.We agree with the reviewer's interpretation. The observed imbalances in late-onset sepsis, transfusions, diuretics, steroids, and longer respiratory support are more likely consequences of pCMV infection or its clinical management (i.e., potential mediators or colliders) rather than pre-infection confounders. To more rigorously control for baseline disease severity that might influence both testing probability and BPD risk, we performed a **sensitivity analysis using a stratified modeling approach**. We constructed a series of multivariable logistic regression models to account for confounding factors(detailed in our response to Comments 2 & 3 and presented in Table 5). Following the reviewer's advice, we've updated the discussion section to acknowledge the limitation of potential residual confounding from unmeasured severity indicators. We've also softened the causal language, emphasizing a strong, independent association instead of definitive causation.

*2) I think using a limited set of variables to perform the match with is justified in the sense that GA, BW, sex likely contribute the most to an infant's severity of illness and risk of BPD. However, did the authors consider whether additional early clinical factors available prior to CMV testing (i.e., early IMV, surfactant use, Apgar scores, early sepsis evaluations) should have been included?*

**Response:**

**Thank you for the valuable suggestion. We agree that controlling early clinical factors through matching strengthens causal inference. Therefore, in our multivariable model 3, we incorporated additional early clinical factors that are available prior to CMV testing, such as early IMV, surfactant administration, Apgar scores, and early sepsis evaluations. This model aims to better control for confounding due to early disease severity. As shown in Table 4, even after adjusting for these factors, the association between pCMV infection and BPD (2018/2019 criteria) remains significant with an enhanced effect size (aOR 11.05, 95% CI 4.00-30.52), supporting the independence of this association. We have clarified the model construction strategy in the methods :** Multivariable logistic regression models were subsequently conducted that accounted for possible confounding factors: Model 1 (crude); Model 2, which was adjusted for matching variables (GA, BW, sex, SGA status); Model 3, which was additionally adjusted for early clinical variables available prior to the pCMV diagnosis (mode of delivery, premature rupture of membranes, 1- and 5-min Apgar scores, early IMV, surfactant administration, early-onset sepsis); Model 4, which included all variables from Model 3 plus potential mediators or later-occurring clinical factors (late-onset sepsis, red blood cell transfusion, receipt of diuretics, receipt of glucocorticoids); and Model 5, which included all variables from Model 4 plus treatment for pCMV. (P7 L122-129**) and results sections of the revised manuscript.**

*3) Several adjusted covariates including transfusions, diuretics, glucocorticoids, and late-onset sepsis may occur after pCMV infection and therefore function as effect modifiers or mediators and not confounders. Globally, we do this all of the time in neonatology studies given the nature of the research and difficulty of parsing out time-varying covariates in our infants with outcomes at one point in time based on clinical diagnostic criteria as opposed to a pathologic diagnosis irrespective of time. However, including these variables may bias effect estimates. Did the authors consider a sensitivity analysis to only include variables that would occur before pCMV diagnosis?*

**Response:**

**We express our gratitude to the reviewer for their insightful critique regarding our methodology. In response to the reviewer's suggestions and to evaluate potential biases, we have undertaken the following analyses:**

**As previously noted, Model 3 incorporates only those variables available prior to the diagnosis of pCMV, aligning with the reviewer's recommendation for a sensitivity analysis that includes only prior variables. In this model, the significant association between pCMV and BPD persists, and the effect size remains robust. Model 4 encompasses all variables, including potential intermediate variables such as transfusion and diuretics. The results demonstrate that the association remains significant, with effect sizes comparable to those observed in Model 3. For instance, the adjusted odds ratio (aOR) for BPD in 2018 is 11.05 in Model 3 and 10.86 in Model 4. This suggests that, despite the presence of mediating pathways, the overall effect of pCMV on BPD remains significant. We cautiously avoid interpreting the adjusted estimates in Model 4 as estimates of direct effects and instead view them as supplementary analyses demonstrating the strength of the association.We have incorporated additional explanations in the discussion section of the revised manuscript to elucidate these findings.We believe that by clearly presenting the results of Model 3 (prior variable model) and Model 4 (full variable model), we have addressed the concerns raised regarding bias from intermediate variables.Table 4 and Methods in** P7 L122-129 **)** *Minor concerns:
1) The manuscript currently emphasizes that newer definitions “better capture” the severity attributable to pCMV. Although plausible, alternative explanations should be acknowledged such as the 2001 NICHD definition misclassifying infants on noninvasive respiratory support but low FiO2 as mild BPD. Alternatively, modern criteria classify infants based on respiratory support modality, which may track with prolonged illness trajectories regardless of pCMV etiology. Differences may reflect criteria sensitivity, not necessarily pathophysiologic differences. The discussion could be expounded upon with this nuance.*
**Response:**

**We express our gratitude to the reviewer for their valuable insight. We concur that the observed variations in associations across different BPD definitions may indeed be attributed to differences in criteria sensitivity and structure, rather than a pathophysiology specific to pCMV. Accordingly, we have revised the pertinent paragraph in the Discussion section to address this nuance. This revision incorporates the reviewer's proposed alternative explanations concerning potential misclassification by the 2001 criteria and the inherent capability of contemporary criteria to monitor prolonged respiratory morbidity irrespective of its etiology:** However, the overall diagnostic rate did not differ significantly between groups. This suggests that pCMV infection may exacerbate BPD severity. However, alternative interpretations must be considered. The observed differences may be attributable, at least in part, to inherent variations in the sensitivity of the diagnostic criteria themselves rather than exclusively indicating a distinct pathophysiology specific to pCMV. The 2001 definition, which relies on FiO₂ levels assessed at a fixed time point, may misclassify infants on substantial non-invasive support with low oxygen concentrations as merely having "mild" disease. Conversely, the 2018 NICHD and 2019 NRN criteria, which assess severity based on type of respiratory support, are inherently more sensitive at identifying prolonged respiratory insufficiency from any cause. Nevertheless, our data substantiate the significant role of pCMV in driving severe diseases. The robust and independent association between pCMV and the highest disease severity (Grades II–III/2–3) within modern frameworks indicates that pCMV is a key contributor to severe, protracted respiratory morbidity. This finding aligns with that of a recent report by Tran et al. [35], who identified an elevated risk of moderate to severe BPD under the 2001 definition. The 2018 NICHD and 2019 NRN criteria are more stringent and better predictors of long-term adverse pulmonary outcomes [36]. The strong association of pCMV with BPD under these definitions further suggests that pCMV not only exacerbates disease severity but may also contribute to a poorer long-term prognosis. (P21 **L228-243)**
*2) For the tables, please use a consistent case. For example Table 2, capitalize the first letter of the first word throughout. There are also numerous instances of variability in the authors referring to BPD severity as mild, moderate, or severe or Grade I-III (or 1-3 depending on the definition). Being consistent throughout would help the uninitiated reader.*

**Response:**

**We appreciate your feedback and thank the suggestion. We have revised all tables to ensure consistent capitalization by capitalizing the initial letter of each entry.We intentionally used the original severity labels to accurately reflect the distinct diagnostic criteria being compared. To aid reader comprehension, we add a clarifying note in the Methods section(Table 1 annotation), and ensure that the text explicitly references the relevant criterion when discussing each severity grade.**
