# Supplementary material for: Postnatal cytomegalovirus infection and bronchopulmonary dysplasia in very low birth weight infants: Influence of diagnostic criteria and viral load in a propensity score–matched cohort study
Source: PLoS One. 2026 Apr 15;21(4):e0346905. doi: 10.1371/journal.pone.0346905 (PMC13082651; doi:10.1371/journal.pone.0346905)
Supplement: Attachment — Submitted filename: Response_to_Reviewers_auresp_2.docx [file pone.0346905.s004.docx]

**Response Letter**

**Manuscripts number:** PONE-D-25-54667R1

**Title:** Postnatal cytomegalovirus infection and bronchopulmonary dysplasia in very low birth weight infants: Influence of diagnostic criteria and viral load in a propensity score-matched cohort study

Dear Editors and Reviewers,

We would like to express our sincere gratitude for your thoughtful and constructive comments on our manuscript. These comments are highly valuable and instrumental for revising and improving our paper, and offer significant guidance for our research. In the following section, we provide detailed responses to the comments. Specifically, the comments are presented in *italics*, our responses are shown in red, and changes to the manuscript are marked in yellow.

*Reviewer #2: ...The authors may wish to consider whether a different matching approach would provide improved balance and interpretability. For example, matching on gestational age, birth weight, sex, and birth year alone would capture the primary baseline determinants of prematurity-related risk while avoiding potential redundancy introduced by including SGA simultaneously with gestational age and birth weight. SGA could then be incorporated as a covariate in adjusted models rather than as a matching variable. Alternatively, if the authors prefer to retain the current matching specification, the residual imbalance in SGA should be explicitly acknowledged in the manuscript. Specifically, the manuscript should note that SGA remained imbalanced after matching (SMD >0.20), discuss the potential implications for interpretation of the findings, and clarify that this may reflect the close relationship between gestational age, birth weight, and SGA status. Addressing this issue, either by reconsidering the matching specification or by more explicitly acknowledging the remaining imbalance and its implications, would strengthen the methodological clarity of the manuscript.*

Response：Thank you for this valuable comment. The insights regarding our propensity score matching strategy are highly valued and have helped us improve the clarity and rigor of our work. We agree that the post-matching standardized mean difference for SGA (SMD > 0.20) indicates a residual imbalance, despite adequate balance on GA and BW. Following your constructive suggestion, we have explicitly acknowledged this limitation. Accordingly, we have made the following revisions to the manuscript:

In the Results section (following Table 2), we have now explicitly noted the residual imbalance in SGA after matching: The post-matching diagnostic assessments indicated that the majority of variables were well-balanced. However, a residual imbalance in SGA status remained (SMD = 0.228). (P8 L140-142)

In the Discussion section (Limitations), we have added discuss to this issue: Second, an imbalance persisted in the SGA following the matching process (SMD = 0.228), potentially attributable to the somewhat over-constrained simultaneous matching for SGA, GA, and BW, in conjunction with the relatively small size of the exposed cohort (n = 74). Although this imbalance was accounted for in the regression models, residual confounding may still be present. Nevertheless, the effect estimates across various models were consistent, indicating that the impact of such confounding is likely minimal. (P23 L278-283)

We believe that these revisions adequately address the concerns you raised and enhance the clarity and transparency of our study.
